# Oxidative Insults and Mitochondrial DNA Mutation Promote Enhanced Autophagy and Mitophagy Compromising Cell Viability in Pluripotent Cell Model of Mitochondrial Disease

**DOI:** 10.3390/cells8010065

**Published:** 2019-01-17

**Authors:** Dar-Shong Lin, Yu-Wen Huang, Che-Sheng Ho, Pi-Lien Hung, Mei-Hsin Hsu, Tuan-Jen Wang, Tsu-Yen Wu, Tsung-Han Lee, Zo-Darr Huang, Po-Chun Chang, Ming-Fu Chiang

**Affiliations:** 1 Department of Pediatrics, Mackay Memorial Hospital, Taipei 10449, Taiwan; pedcsho@gmail.com; 2 Department of Medicine and Institute of Biomedical Sciences, Mackay Medical College, New Taipei 25245, Taiwan; 3 Department of Medical Research, Mackay Memorial Hospital, Taipei 10449, Taiwan; wendyhuang1219@gmail.com (Y.-W.H.); linws@mmh.org.tw (T.-Y.W.); randy.b746mmh@gmail.com (T.-H.L.); darr.9475@mmh.org.tw (Z.-D.H.); 4 Department of Pediatric Neurology, Kaohsiung Chang Gung Memorial Hospital, and Chang Gung University College of Medicine, Kaohsiung 88301, Taiwan; flora1402@cgmh.org.tw (P.-L.H.); a03peggy@cgmh.org.tw (M.-H.H.); 5 Department of Laboratory Medicine, Mackay Memorial Hospital, Taipei 10449, Taiwan; dj.wang@mmh.org.tw; 6 Department of Information Technology, Mackay Memorial Hospital, Taipei 10449, Taiwan; f1481101@gmail.com; 7 Department of Neurosurgery, Mackay Memorial Hospital, Taipei 10449, Taiwan; 8 Mackay Medicine, Nursing and Management College, Taipei 11260, Taiwan; 9 Graduate Institute of Injury Prevention and Control, Taipei Medical University, Taipei 11031, Taiwan

**Keywords:** mitochondrial diseases, MELAS, iPS cells, autophagy, mitophagy

## Abstract

Dysfunction of mitochondria causes defects in oxidative phosphorylation system (OXPHOS) and increased production of reactive oxygen species (ROS) triggering the activation of the cell death pathway that underlies the pathogenesis of aging and various diseases. The process of autophagy to degrade damaged cytoplasmic components as well as dysfunctional mitochondria is essential for ensuring cell survival. We analyzed the role of autophagy inpatient-specific induced pluripotent stem (iPS) cells generated from fibroblasts of patients with mitochondrial encephalomyopathy, lactic acidosis, and stroke-like episodes (MELAS) with well-characterized mitochondrial DNA mutations and distinct OXPHOS defects. MELAS iPS cells recapitulated the pathogenesis of MELAS syndrome, and showed an increase of autophagy in comparison with its isogenic normal counterpart, whereas mitophagy is very scarce at the basal condition. Our results indicated that the existence of pathogenic mtDNA alone in mitochondrial disease was not sufficient to elicit the degradation of dysfunctional mitochondria. Nonetheless, oxidative insults induced bulk macroautophagy with the accumulation of autophagosomes and autolysosomes upon marked elevation of ROS, overload of intracellular calcium, and robust depolarization of mitochondrial membrane potential, while mitochondria respiratory function was impaired and widespread mitophagy compromised cell viability. Collectively, our studies provide insights into the dysfunction of autophagy and activation of mitophagy contributing to the pathological mechanism of mitochondrial disease.

## 1. Introduction

Mitochondria are double-membrane-bound organelles with two mitochondrial compartments including the intermembrane space and the matrix. Mitochondria have a pivotal role in cell energy homeostasis which is of relevance to cellular physiology. The electron transport system and the adenosine triphosphate (ATP) sythase complex located on the inner mitochondrial membrane and enzymes in the matrix play a vital role in the proceeding of ATP production via the citric acid cycle, fatty acid oxidation and oxidative phosphorylation system (OXPHOS) [1]. Mitochondria DNA (mtDNA), located in the matrix, contains 37 genes encoding 13 proteins, 22 tRNAs, and two rRNAs [2]. The 13 mitochondrial genes encode 13 polypeptide subunits of the respiratory chain complexes of the oxidative phosphorylation system for cellular energy production, while the remaining 79 structural OXPHOS subunits are encoded by the nuclear genome [3].

Mutations of mtDNA result in OXPHOS defects which are characterized by a broad spectrum of clinical manifestations and multi-system involvement [4,5,6]. Of note, more than 50% of mtDNA mutations are located in 22 tRNA genes [7]. Whereas the A3243G mutation in the mitochondrial *tRNA^Leu(UUR)^* gene (MT-TL1) is one of the most common mtDNA mutations and can give rise to mitochondrial encephalomyopathy, lactic acidosis, and stroke-like episodes (MELAS), as well as maternally inherited diabetes and deafness [8,9]. High levels of A3243G mutation cause severe assembly defects of respiratory chain complexes I and IV leading to an impaired biogenesis, which is characterized with an increase in glycolytic flux, lactate, and reactive oxygen species (ROS) production, as well as a decrease in mitochondrial membrane potential and ATP synthesis [10,11,12]. Furthermore, the failure to switch substrate utilization from glucose oxidation to fatty acid oxidation in response to energy deficiency is mediated by 5′-adenosine monophosphate-activated protein kinase (AMPK) and may contribute to the development of the clinical phenotype [12].

Under normal conditions, cellular ROS can be scavenged by the antioxidant system to re-establish or maintain redox homeostasis. Nonetheless, cell damage occurs upon the failure of the cell’s antioxidant system, either exceeding its capacity or being less active, to purge the accumulation of ROS. The prevalence or accumulation of damaged organelles and aggregated protein within the cells can trigger the activation of cell death pathway, which has a deleterious impact upon tissues, organisms, and biological systems, and leads to the development of aging and various diseases. To maintain the cellular homeostasis and promote cell survival, the compromised cellular components are degraded by the process of autophagy into basic molecules for recycling in biosynthetic or catabolic processes [13]. Dysregulation or impairment of autophagy has been implicated in aging, infection, cancer, and degenerative diseases. To this date, studies of autophagy upon mitochondrial dysfunction induced by mtDNA A3243G mutation were limited to fibroblasts and cybrids; these studies showed controversial results and warrant more studies to unveil the mechanism [14,15,16]. Recently, the use of patient-specific induced pluripotent stem (iPS) cells enable to model of a unique human disease and contributed to a better understanding of its pathogenesis, to the discovery of new drugs, and to the development of novel therapy. In the present study, MELAS iPS cells harboring high levels of the mitochondrial A3243G mutation showed elevated levels of autophagy and scarcity of mitophagy in comparison with its normal counterpart harboring an isogenic background. Oxidative insults induced a marked increase of bulk macroautophagy, autophagic flux dysfunction, and broad activation of mitophagy, and led to compromised cell viability in the MELAS iPS cells.

## 2. Material and Methods

### 2.1. Generation of iPS Cell and Culture

In compliance with the Declaration of Helsinki of the World Medical Association, informed consent form was approved by the Institutional Review Board of Mackay Memorial Hospital and was obtained from the patient before any investigation of this study. Primary skin fibroblasts derived from patient with MELAS syndrome harboring mtDNA A3243G mutation were cultured in standard Dulbecco’s modified Eagle medium (DMEM; Invitrogen, Carlsbad, CA, USA), supplemented with 10% (*v*/*v*) fetal bovine serum (FBS; Life Technologies, Grand Island, NY, USA) and 1% penicillin G/streptomycin sulfate, in a humidified atmosphere of 5% (*v*/*v*) CO_2_ at 37 °C. Reprogramming of fibroblasts was carried out with a modified, non-transmissible form of Sendai virus according to the manufacturer’s protocol (CytoTune-iPS Reprogramming Kit, Thermo Fisher Scientific, Waltham, MA, USA). After transduction, undifferentiated iPS colonies were isolated manually and propagated in Essential 8™ Medium (Thermo Fishers Scientific) on vitronectin-coated culture dishes. When indicated, the iPS cells were plated at a density of 400,000 cells per well of a six-well cell culture plate overnight. The next day, iPS cells were treated with or without 2 μM Carbonyl cyanide m-chlorophenylhydrazone (CCCP) for 4 h in the absence or presence of Bafilomycin (BAF).

### 2.2. Immunohistochemistry

Cultured cells were fixed with 4% paraformaldehyde in Phosphate-buffered saline (PBS) for 20 min at room temperature, rinsed three times with PBS, then permeabilized with 0.2% Triton X-100 in PBS for 10 min. Cells were blocked with 10% goat serum (Sigma, St Louis, MO, USA) for 1 h at room temperature, incubated with primary antibodies for 1 h at room temperature, rinsed 3 times with PBS, then incubated with secondary antibodies (1:500, Molecular Probes, Invitrogen) for 1 h at room temperature. The cells were washed three times with PBS, counterstained with 4′,6-diamidino-2-phenylindole (DAPI), and visualized under fluorescence microscope. Primary antibodies were: Lin-28 Homolog A (LIN28, 1:100, GeneTex, Irvine, CA, USA), Octamer-binding transcription factor 4 (OCT4, 1:100, Genetex), stage-specific embryonic antigen (SSEA4, 1:100, Thermo Fisher Scientific) and carbonhydrate TRA-1-60 epitope (1:100, Thermo Fisher Scientific).

### 2.3. Live Cell Oxygen Consumption

Bioenergetic profiles were determined using the XF24 extracellular flux analyzer (Seahorse Biosciences, Santa Clara, CA, USA). iPS cells were seeded at a density of 40,000 cells per well of a XF24 cell culture microplate and maintained in Essential 8™ Medium overnight in a humidified atmosphere of 5% (*v*/*v*) CO_2_ at 37 °C. Before assay, iPS cells were equilibrated in unbuffered DMEM medium (Life Technologies) supplemented with 25 mM glucose, 1 mM sodium pyruvate, 2 mM L-Glutamine and transferred to a non-CO_2_ incubator for 1 h before measurement. Oxygen consumption rate (OCR) was measured with sequential injections of different concentration of CCCP and each 0.4 μM of rotenone/antimycin A.

OCR was increased immediately after the addition of CCCP, which enabled the measurement of maximal mitochondrial respiration, and was completely abolished after the inhibition of OXPHOS by injection of rotenone/antimycin A. Each plotted value of real-time assessment of mitochondrial respiration was represented as percentage of basal OCR. Results were presented as mean ± SEM.

### 2.4. Western Blot Analysis

The cells were harvested and lysed with Radio-Immune Precipitation Assay (RIPA) lysis buffer. The supernatant was collected after centrifugation of the cell lysate and the protein was measured using the bicinchoninic acid (BCA) protein assay (Thermo Fisher Scientific). An aliquot of 20 μg of whole cell extracts was separated by sodium dodecyl sulfate–polyacrylamide gel electrophoresis (SDS-PAGE) using 10% or 12% polyacrylamide gels. After electrophoresis, proteins were transferred to a piece of Polyvinylidene difluoride membrane (PVDF), which was immunoblotted for Microtubule Associated Protein Light Chain 3 (LC3, 1:1000, Sigma), Complex I subunit Ubiquinone Oxidoreductase Subunit B8 (NDUFB8), Complex II subunit Succinate Dehydrogenase Complex Iron Sulfur Subunit B (SDHB), Complex III subunit Ubiquinol-Cytochrome C Reductase Core Protein 2 (UQCRC2), Complex IV subunit Mitochondrially Encoded Cytochrome C Oxidase II (MTCO2), Complex V subunit ATP Synthase F1 Subunit Alpha (ATP5A), porin (1:1000; Abcam, Cambridge, MA, USA), Myelocytomatosis oncogene cellular homolog (c-Myc), Kruppel Like Factor 4 (Klf4), Lin-28 Homolog A (Lin28A), Octamer-binding transcription factor 4 (Oct4), and SRY-Box 2 (Sox2) (1:1000, Genetex) for 2 h at room temperature. The PVDF membrane was then washed, probed with horseradish peroxidase-conjugated secondary antibodies for 1 h at room temperature, washed again, and then visualized by enhanced chemiluminescence (GE, Healthcare Life Sciences, Chicago, IL, USA). 

### 2.5. Detection of Autophagosomes, Autolysosomes and Mitochondria

In brief, after treatment of the iPS cells with or without 2 μM CCCP for 4 h, the cells were stained with CYTO-ID Green detection reagent (Enzo Life Science, Farmingdale, NY, USA), LysoTrakcer Red (Thermo Fisher Scientific), MitoTracker Green (Thermo Fisher Scientific), respectively, and counterstained with Hoechst 33342, according to the manufacturer’s protocol. Images were obtained using a fluorescent microscope. To quantize the levels of autophagosomes, the iPS cells, in the presence or absence of CCCP, were trypsinized, centrifuged, washed, and resuspended in CYTO-ID assay buffer. After staining them with CYTO-ID Green detection reagent, the iPS cells were centrifuged, washed, resuspended in assay buffer, and analyzed on a flow cytometer.

### 2.6. Reactive Oxygen Species (ROS) Detection Using a Fluorescent Agent MitoSox

Superoxide anion levels were measured using the fluorescent dye MitoSox Red (Thermo Fisher Scientific). The iPS cells were treated with MitoSox Red (5 μM) for 10 min at 37 °C in the dark, washed, and counterstained with DAPI for 5min. Images were obtained using a fluorescent microscope. To qualify the levels of reactive oxygen species, the iPS cells were seeded on a 96-well plate and treated with or without 2 μM CCCP for 4 h, then incubated with MitoSox Red (5 μM) for 10 min at 37 °C in the dark, and washed with 0.1% FBS in HHBS. The fluorescent intensity was read with a microplate reader (Infinite M200PRO, TECAN, Mannedorf, Switzerland). All measurements, normalized for number of cells, were presented as mean ± SEM. Cell numbers were quantified by the CyQUANT cell proliferation assay kit (Molecular Probes, Invitrogen).

### 2.7. Measurement of Mitochondrial Membrane Potential

The iPS cells were seeded on a 96-well plate and treated with/without 2 μM CCCP for 1 h, then incubated with tetramethylrhodamine ethyl ester (TMRE, 50 nm; Thermo Fisher Scientific) for 20 min at 37 °C in the dark, and washed with 0.2% BSA in PBS. The fluorescent intensity was read with a microplate reader, and the images were obtained using a fluorescent microscope. All measurements, normalized for number of cells, were presented as mean ± SEM. Cell numbers were quantified by the CyQUANT^®^ cell proliferation assay kit.

### 2.8. Detection of Intracellular Calcium Mobilization

The iPS cells were seeded on a 96-well plate and treated with/without 2 μM CCCP for 4 h, incubated with Fluo-8 calcium assay kit (AAT Bioquest, Sunnyvale, CA, USA) for 1 h at 37 °C, 5% CO_2_ in incubator, and washed with Hank’s Buffer with HEPES (HHBS) according to the manufacturer’s protocol. The fluorescent intensity was read with a microplate reader. All measurements, normalized for number of cells, were presented as mean ± SEM. Cell numbers were quantified by the CyQUANT cell proliferation assay kit.

### 2.9. Measurement of Intracellular ATP Content

The intracellular ATP content was measured by the ATPlite^TM^ Luminescence Assay system (PerkinElmer, Boston, MA, USA) according to the instruction of manufacturer. Briefly, the iPS cells were seeded on a 96-well plate and treated with/without 2 μM CCCP for 4 h, washed with PBS, mixed with mammalian cell lysis solution, shacked for 5 min at 800× *g*, mixed with substrate solution, and shacked for 5 min at 800× *g* to release the intracellular ATP. The 96-well culture plate was dark-adapted for 10 min. Luminescence intensity from each well was measured using an Infinite 200 pro plate reader (TECAN). The intracellular ATP content was normalized by the cell number. Cell numbers were quantified by the CyQUANT cell proliferation assay kit.

### 2.10. Cell Viability Assay

The iPS cells were seeded on a 96-well plate and treated with or without 2 μM CCCP for 4 h in a humidified atmosphere of 5% (*v*/*v*) CO_2_ at 37 °C. Cell numbers were quantified by the CyQUANT cell proliferation assay kit. Cells were incubated with CyQUANT dye at 37 °C for 30 min and fluorescence intensity was measured on a plate reader at OD = 530 nm.

### 2.11. Statistical Analysis

All data were obtained from at least three independent experiments and results were expressed as the mean ± SEM, unless stated otherwise. An ANOVA test was used for multiple comparisons. When *p*-values were less than 0.05, they were considered significant. 

## 3. Results

### 3.1. Identifying Cell Surface Markers and Respiratory Complexes for the iPS Cells 

**Figure 1:**
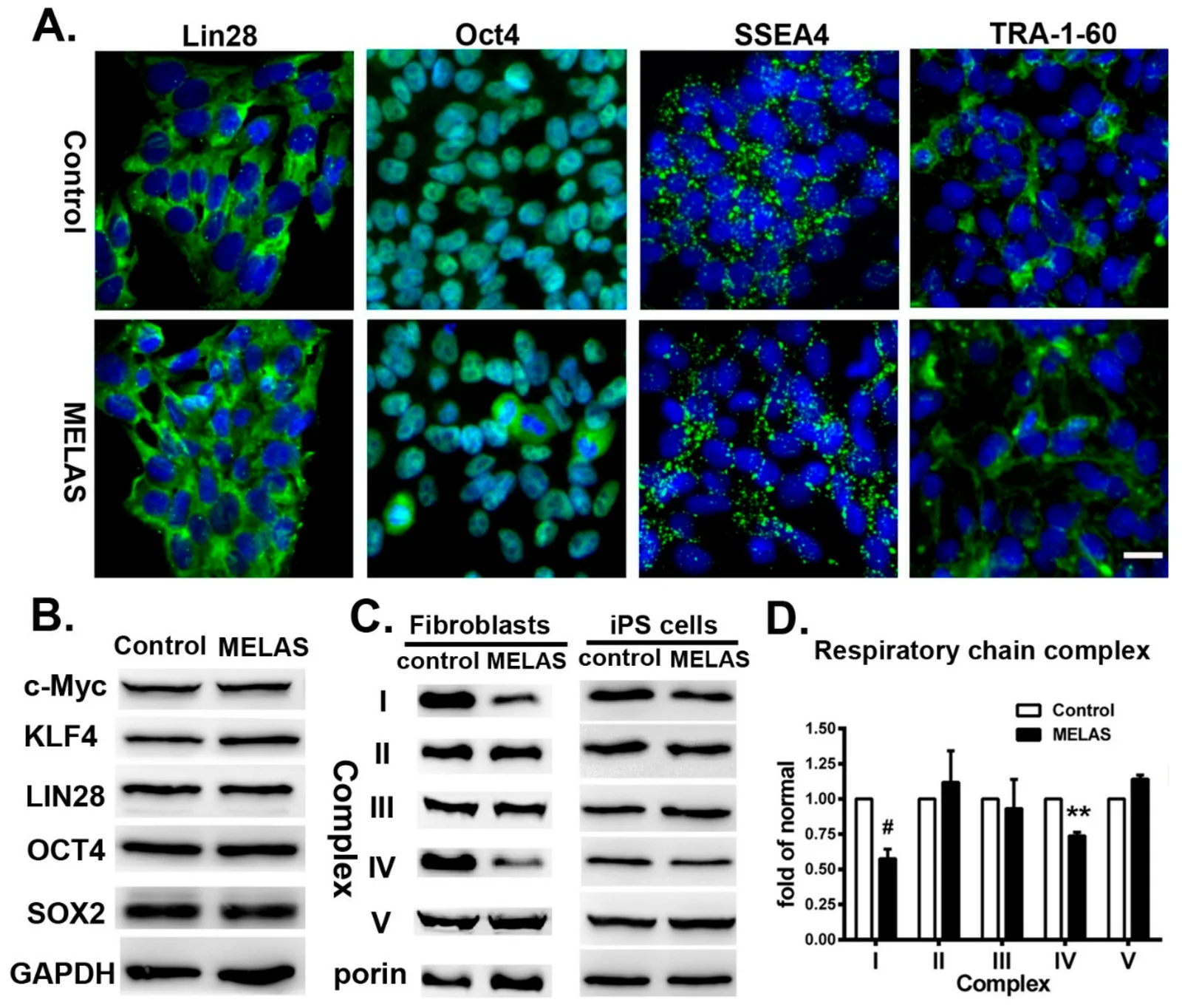
Induced pluripotent stem (iPS) cells derived from fibroblasts of mitochondrial encephalomyopathy, lactic acidosis, and stroke-like episodes (MELAS) syndrome patient. (**A**) Immunocytochemical analyses showed positive expression of pluripotency markers Lin28, Oct4, SSEA4 and Tra-1-60 on both control and MELAS iPS cells. (**B**) Western blots showing the expression of c-Myc, Klf4, Lin28A, Oct4, Sox2 in iPS cells. (**C**) Western blots showing the expression of respiratory complexes in fibroblasts and iPS cells, respectively. (**D**) Quantification of respiratory chain complex proteins in iPS cells represented as fold of normal. All measurements are expressed as mean values ± SEM, n = 3–5. Comparisons with the control cells were performed by the ANOVA-test. ** *p* < 0.01, # *p* < 0.001, versus control. Scale bar: 20 μm.

We reprogrammed fibroblasts from a seven year-old boy with MELAS syndrome harboring 95% mtDNA A3243G mutation to iPS cells using retroviral vectors expressing Oct4, Klf4, Sox2, and c-Myc [12,17]. Mutation segregation occurred in individual iPS cell lines generated from A3243G fibroblasts. Both isogenic iPS cell lines carried 85% heteroplasmy (MELAS iPS) and undetectable A3243G mutation (control iPS), respectively, and showed expression of pluripotent markers (Figure 1A,B). It has been noted that cells with a proportion of pathogenic mitochondrial tRNA mutation lower than 85% to 90% maintains its normal physiological function and phenotype [18]. The MELAS iPS cells demonstrated deficiency of respiratory complexes I and IV in line with its parental fibroblasts (Figure 1C,D) [12].

### 3.2. Enhanced Flux of Autophagy

**Figure 2:**
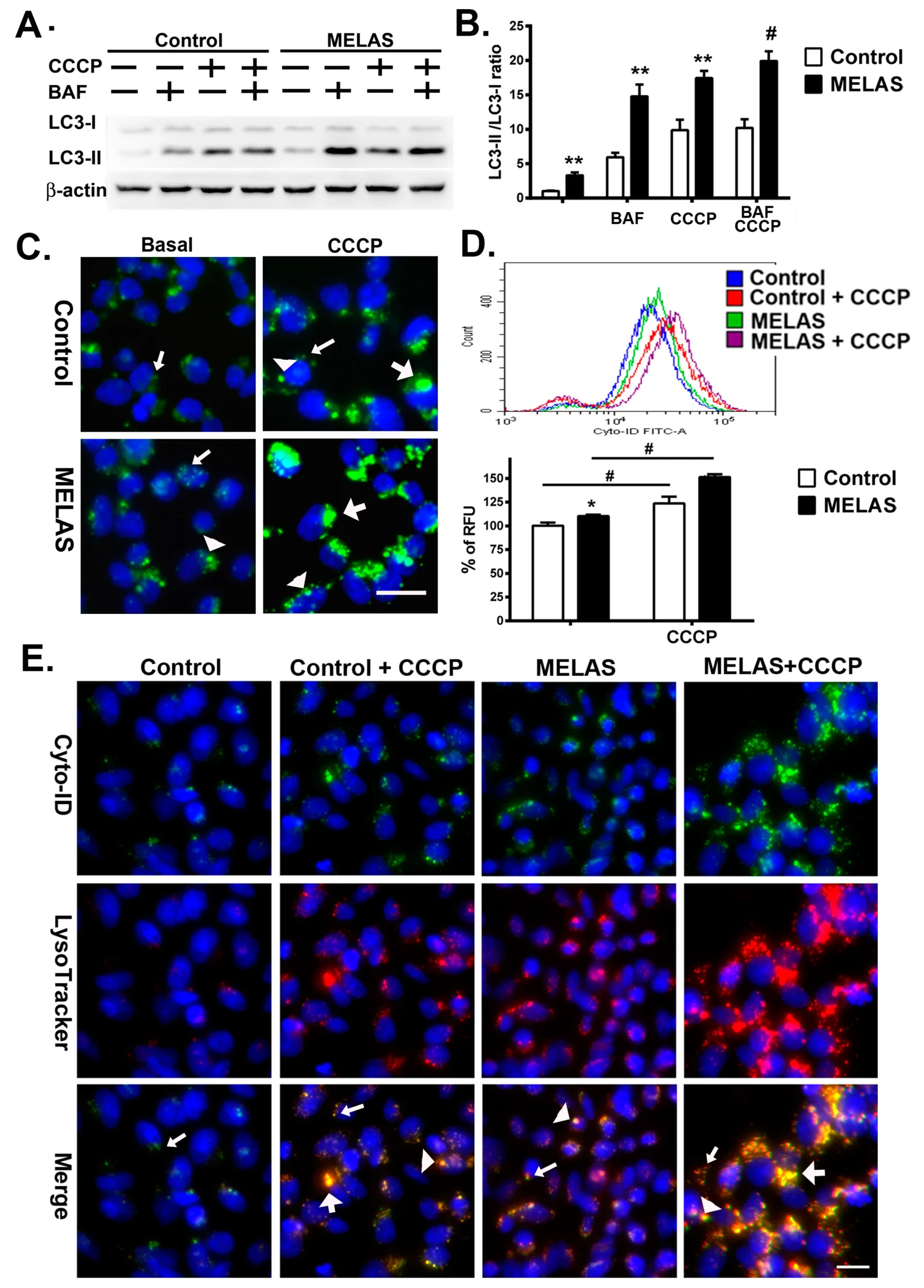
Increase of autophagic flux in MELAS iPS cells. (**A**) Western blots of LC3 expression of iPS cells in the presence or absence of bafilomycin (BAF) and Carbonyl cyanide m-chlorophenylhydrazone (CCCP), respectively, for the study of autophagic flux. (**B**) Quantification of LC3 levels normalized for β-actin. (**C**) Microscopy fluorescence images showing autophagosomes stained by Cyto-ID green dye. Small arrow indicates small puncta. Arrow head indicates large puncta. Large arrow indicates enlarged puncta. (**D**) Representative flow cytometry histogram and quantification of Cyto-ID green fluorescence levels normalized for number of cells. (**E**) Microscopy fluorescence images showing autolysosomes by colocalization of Cyto-ID green dye and LysoTracker Red. Small arrow indicates small puncta. Arrow head indicates large puncta. Large arrow indicates enlarged puncta co-localized with Cyto-ID green and LysoTracker Red. All measurements are expressed as mean values ± SEM, n = 3–5. * *p <* 0.05, ** *p <* 0.01, # *p <* 0.001, versus control. RFU: relative fluorescence unit. Scale bar: 20 μm.

Autophagy is considered to be a dynamic process comprising the formation of autophagosomes, autolysosomes, and the degradation of autophagic substrates. Evaluation of the amount of LC3-II is the most widely used autophagosome marker to correlate the flux of autophagy with the number of autophagosomes. CCCP is a protonophore which uncouples oxidative phosphorylation, induces ROS and depolarizes mitochondrial membrane potential, thus, triggering mitophagy and bulk autophagy [19]. Researchers have typically challenged cells with CCCP to initiate the autophagy for assessing the oxidative stress induced autophagic flux and mitophagy. Bafilomycin is commonly used to inhibit autophagy by targeting lysosomes. To determine the level of autophagy in MELAS cells, the classical autophagy markers LC3-I and LC3-II were analyzed following treatment with or without CCCP in the absence or presence of bafilomycin. Western blot analysis revealed a significant increase of LC3II/LC3I ratio in MELAS iPS cells compared to control iPS cells at basal level (2.65 versus 1.0), treatment with either CCCP (15.90 versus 8.33) or bafilomycin (11.32 versus 4.90) alone, and a combination of CCCP and bafilomycin (17.10 versus 8.40), respectively (Figure 2A,B). The treatment of bafilomycin in control and MELAS iPS cells led to a significant increase in the LC3-II/LC3-I ratio, suggesting that the lysosomal flux is normal in both iPS cell lines. Treatment with CCCP in MELAS iPS cells led to a significantly higher surge of LC3-II/LC3-I ratio in comparison with control iPS cells. These results indicated an enhanced autophagy flux in MELAS iPS cells at basal condition and upon oxidative stress.

### 3.3. Accumulation of Autophagosomes

When considering the ectopic localization of LC3-II on non-autophagosome structures, the expression levels of LC3-II at a specific time does not necessarily represent the overall autophagic activity [20,21]. Thus, to further validate the activity of autophagy flux, iPS cells were live stained with Cyto-ID Green fluorescent dye and autophagosomes are visualized under fluorescent microscopy (Figure 2C) [22]. Both control and MELAS iPS cells showed Cyto-ID stained fluorescent dots at basal condition and in the presence of CCCP. On the other hand, MELAS iPS cells showed more large punctate structures (autophagosomes) in comparison with the control iPS cells upon exposure to CCCP.

Moreover, to quantify the levels of autophagosomes in live cells, the increase in Cyto-ID Green autophagy dye fluorescence signals was determined by flow cytometry [22]. Our results showed a significant increase of autophagosomes in MELAS iPS cells compared to that of control iPS cells at basal condition and in the presence of CCCP (Figure 2D). These observations correlated with the marked increase of LC3 expression assayed by western blotting in MELAS iPS cells (Figure 2A,B), indicating the increase of bulk macroautophagy and accumulation of autophagosome upon oxidative stress.

### 3.4. Accumulation of Autolysosomes

Autophagosomes fuse with lysosomes to form autolysosomes where autophagosomal contents are degraded by proteases within the lysosomes. Fusion between autophagosomes and lysosomes could be visualized by using Cyto-ID Green fluorescent dye and LysoTracker Red to stain autophagosomes and lysosomes, respectively. Under fluorescent microscopy, both iPS cells showed small puncta with positive LysoTracker Red fluorescence in the absence or presence of CCCP, and large puncta staining with LysoTracker Red upon exposure to CCCP (Figure 2E). While MELAS iPS cells demonstrated numerous small puncta and enlarged puncta with LysoTracker Red fluorescence in comparison with the control iPS cells. These findings suggested an increase of lysosomal flux in the MELAS iPS cells. Moreover, the colocalization of Cyto-ID Green fluorescence and LysoTracker Red fluorescence occurred concurrently forming enlarged puncta, and indicated the formation of autolysosome which accumulated more specifically in the MELAS iPS cells upon exposure to CCCP. 

### 3.5. Remarked Increase of ROS

**Figure 3:**
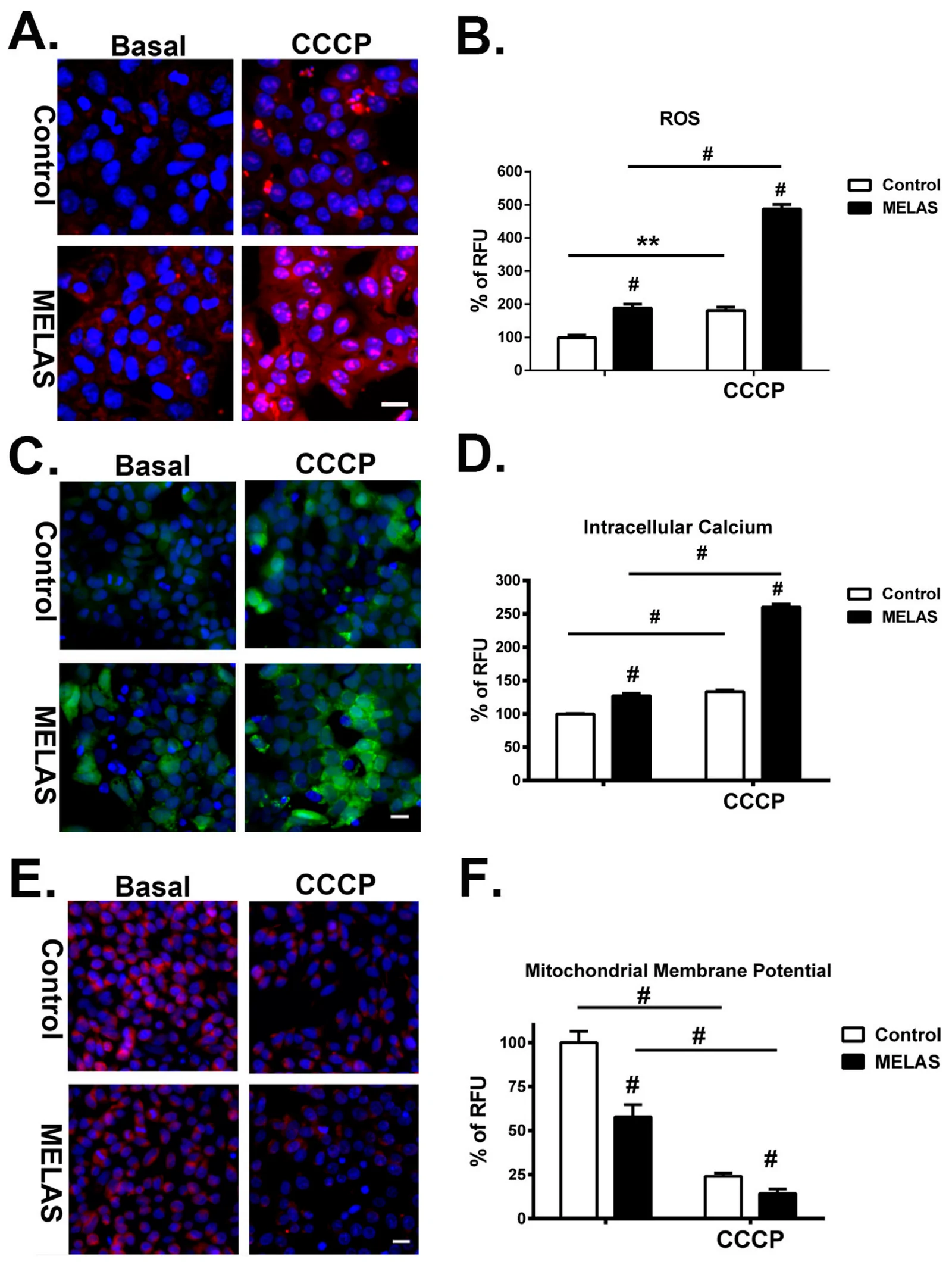
Increase of reactive oxygen species (ROS), intracellular calcium, and mitochondrial depolarization in MELAS iPS cells. (**A**) Representative microscopy fluorescence images showing levels of ROS by staining with MitoSox Red in iPS cells in the presence or absence of CCCP. (**B**) Quantification of ROS levels by analyzing the fluorescence intensity of MitoSox Red. (**C**) Representative microscopy fluorescence images showing levels of intracellular calcium by staining with calcium-sensitive fluorescent dye, fluo-8, in iPS cells in the presence or absence of CCCP. (**D**) Quantification of intracellular calcium levels by analyzing the fluorescence intensity of fluo-8. (**E**) Microscopy fluorescence images showing levels of mitochondrial depolarization by staining with tetramethylrhodamine ethyl ester (TMRE) in iPS cells in the presence or absence of CCCP. (**F**) Quantification of mitochondrial depolarization levels by analyzing the fluorescence intensity of TMRE. All measurements, normalized for number of cells, are expressed as mean values ± SEM, n = 3–5. ** *p* < 0.01, # *p* < 0.001, versus control. RFU: relative fluorescence unit. Scale bar: 20 μm.

The effect of CCCP on mitochondrial ROS production in iPS cells was analyzed by using the fluorescent dye MitoSox Red, which is highly selective for the detection of superoxide in the mitochondria of live cells. Red fluorescence was exhibited when MitoSox Red reagent is readily oxidized by the superoxide in the mitochondria. Furthermore, the oxidation product exhibits a high fluorescence upon binding to nucleic acids. Incubation with CCCP led to a significant increase in MitoSox fluorescence in both iPS cell lines, whereas MELAS iPS cells showed strong intensity of MitoSox fluorescence in the cytoplasm compared to the control iPS cells (Figure 3A). There were also strong fluorescent spots in the nuclei of MELAS iPS cells, without colocalization with fluorescence of MitoTracker Green (data not shown), suggesting the production of superoxide in the nuclei. It has been reported that superoxide radical generated by nuclei as well as by other membranous structures results in DNA base modification and formation of nicks in DNA strands leading to deleterious biological consequences [23]. Furthermore, recent studies indicate that respiratory complex IV deficiency contributes to nuclear and mitochondrial DNA damage [24,25].

Lastly, qualification of the intracellular levels of superoxides revealed a significant increase in both iPS cell lines after incubation with CCCP (Figure 3B). Of note, MELAS iPS cells showed a significantly elevated level of superoxide compared to the control iPS cells at basal condition and in the presence of CCCP, respectively. Additionally, the fold increase of superoxide from basal condition to the exposure to CCCP in MELAS iPS cells was higher than that of control iPS cells.

### 3.6. Overload of Cytoplasmic Calcium Flux

High levels of reactive oxygen and nitrogen species can compromise normal physiological pathways and induce cell death. Oxidative stress increases the calcium influx into the cytoplasm from the extracellular compartment and from the sarco/endoplasmic reticulum through a combination of effects on calcium pumps, exchangers, channels, and binding proteins [26,27]. A rising cytoplasmic concentration of calcium induces calcium influx into the mitochondria and nuclei and leads to down-regulation of mitochondrial metabolism, and subsequently, cell death. The presence of cytosolic calcium was defined and measured by staining with the calcium-sensitive fluorescent dye, fluo-8. Exposure to CCCP induced release of calcium into the cytoplasm in both control and MELAS iPS cells (Figure 3C).It should be noted that, although in the presence of CCCP bright tiny spots were highly abundant within the cytoplasmic region, MELAS iPS cells showed more fluorescent intensity and spots in the cytoplasm.

Quantification of the intracellular calcium concentration demonstrated a significant increase of calcium concentration in both iPS cell lines after incubation with CCCP (Figure 3D). Intriguingly, MELAS iPS cells showed a significantly higher concentration of intracellular calcium than that of the control iPS cells at basal conditions and in the presence of CCCP. Additionally, the increase of intracellular calcium concentration between basal condition and with the exposure to CCCP in MELAS iPS cells was also higher than that of the control iPS cells. These results suggested an enhanced increase of calcium influx into the cytoplasm of MELAS iPS cells in response to oxidative stress.

### 3.7. Robust Depolarization of Mitochondrial Membrane Potential

The increase of ROS and calcium overload in the cytoplasm triggers the opening of the mitochondrial permeability transition pores and leads to dissipation of mitochondrial membrane potential (ΔΨ_m_), reduction of mitochondrial ATP production, and induction of apoptosis and cell death [28,29]. To determine the level of mitochondrial membrane potential, iPS cells were stained with a fluorescent dye, tetramethylrhodamine ethyl ester (TMRE), which redistributes across the cell membrane, accumulates in mitochondria in a voltage-dependent manner, and does not interfere with the cell proliferation and viability [21]. While mitochondrial ΔΨ_m_ collapses in apoptotic cells, the even redistribution of TMRE in the cytosols produced a lower level of fluorescence. Under fluorescent microscopy, we observed a less intense fluorescence of TMRE in MELAS iPS cells compared to that in the control iPS cells at basal conditions. This suggested a loss of mitochondrial membrane potential resulting from OXPHOS defects in MELAS iPS cells (Figure 3E). After incubation with CCCP, TMRE fluorescence declined in both iPS cell lines, while MELAS iPS cells demonstrated a broad loss of fluorescence in comparison with that of control iPS cells. Quantitative measurement of mitochondrial membrane potential by the intensity of emitted TMRE fluorescence further provided a better functional assessment of the dynamic changes to cell activity. We found that ΔΨ_m_ of MELAS iPS cells was 58%of the control iPS at the basal condition and declined to 14% after treatment with CCCP (Figure 3F), whereas ΔΨ_m_ of the control iPS cells declined to 21.9% of its basal condition in the presence of CCCP. These findings were consistent with the observations under fluorescent microscopy. Taken together, the dynamic changes of ΔΨ_m_ were inversely related to the elevation of ROS and intracellular calcium levels. 

### 3.8. Activation of Mitophagy Upon Oxidative Insults

**Figure 4:**
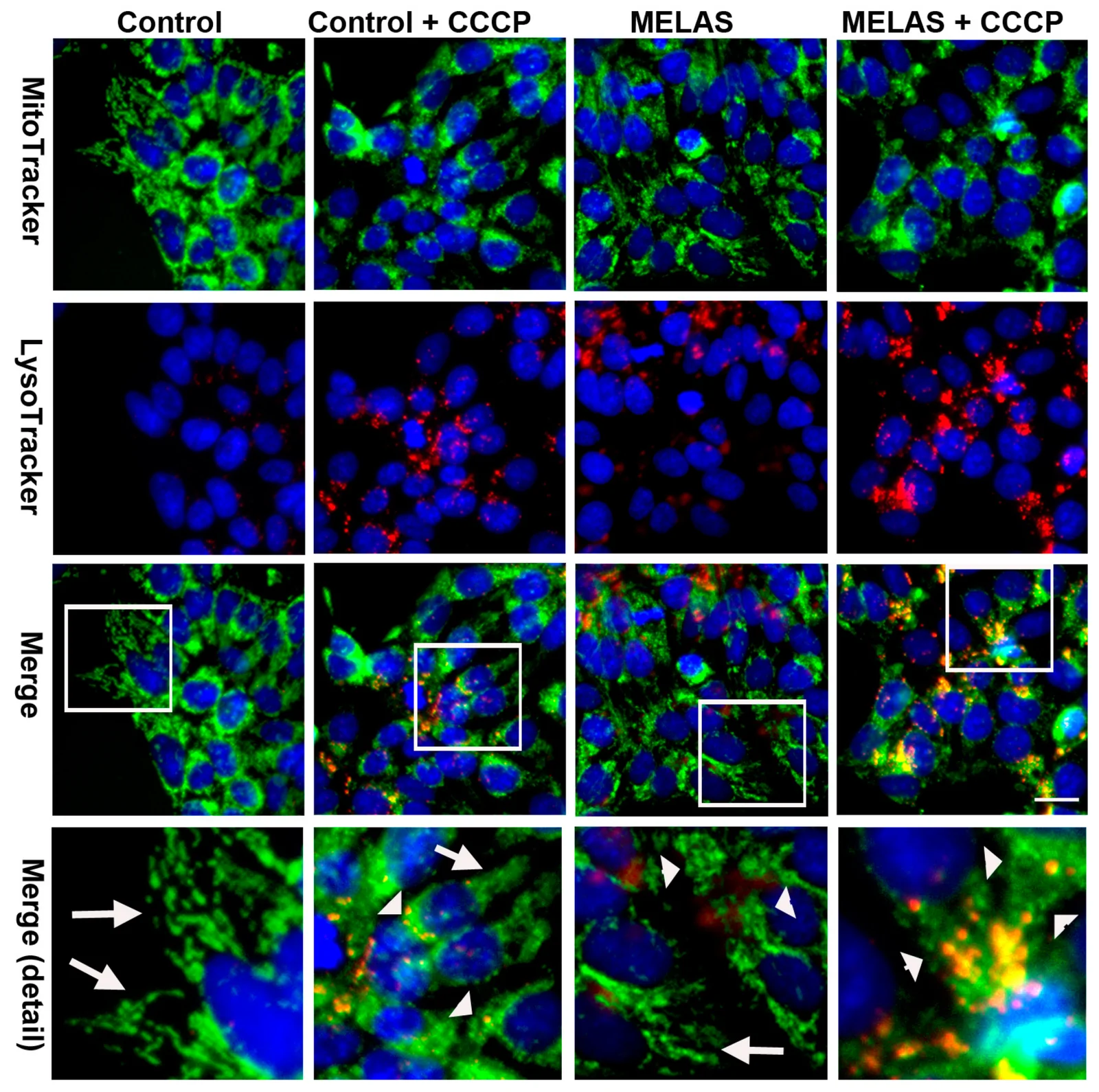
Activation of mitophagy in MELAS iPS cells. Microscopy fluorescence images showing levels of mitophagy after double staining with MitoTracker Green and LysoTracker Red in iPS cells in the presence or absence of CCCP. Colocolization fluorescence of MitoTracker Green and LysoTracker Red are indicative of mitophagy. The lower row shows magnification of the boxed area in the panel. Arrow indicates tubular mitochondria. Arrow head indicates fragmented mitochondria. Scale bar: 20 μm.

To determine whether increased degradation of mitochondria occurred in the MELAS iPS cells during the phase of enhanced autophagy, iPS cells were double stained with LysoTracker Red and MitoTracker Green fluorescent dyes to concurrently label lysosome and mitochondria, respectively. The observation of small fragmented mitochondria was more evident in MELAS iPS cells, whereas a tubular mitochondrial network was clearly distinct in control iPS cells (Figure 4). Moreover, with the LysoTracker Red fluorescence and in comparison with the control iPS cells, the small fragmented mitochondria engulfed within enlarged puncta (autolysosome) broadly visualized in the MELAS iPS cells on exposure to CCCP suggested an enhanced mitophagy degrading damaged mitochondria within autolysosomes. Conversely, mitophagy was very scarce in MELAS iPS and control iPS cells at the basal condition. This observation was consistent with previous findings, whereby selective elimination of mitochondria containing pathogenic mtDNAs is spared in mitochondrial diseases under basal conditions [16]. 

### 3.9. Decrease of Cellular Bioenergetics

**Figure 5:**
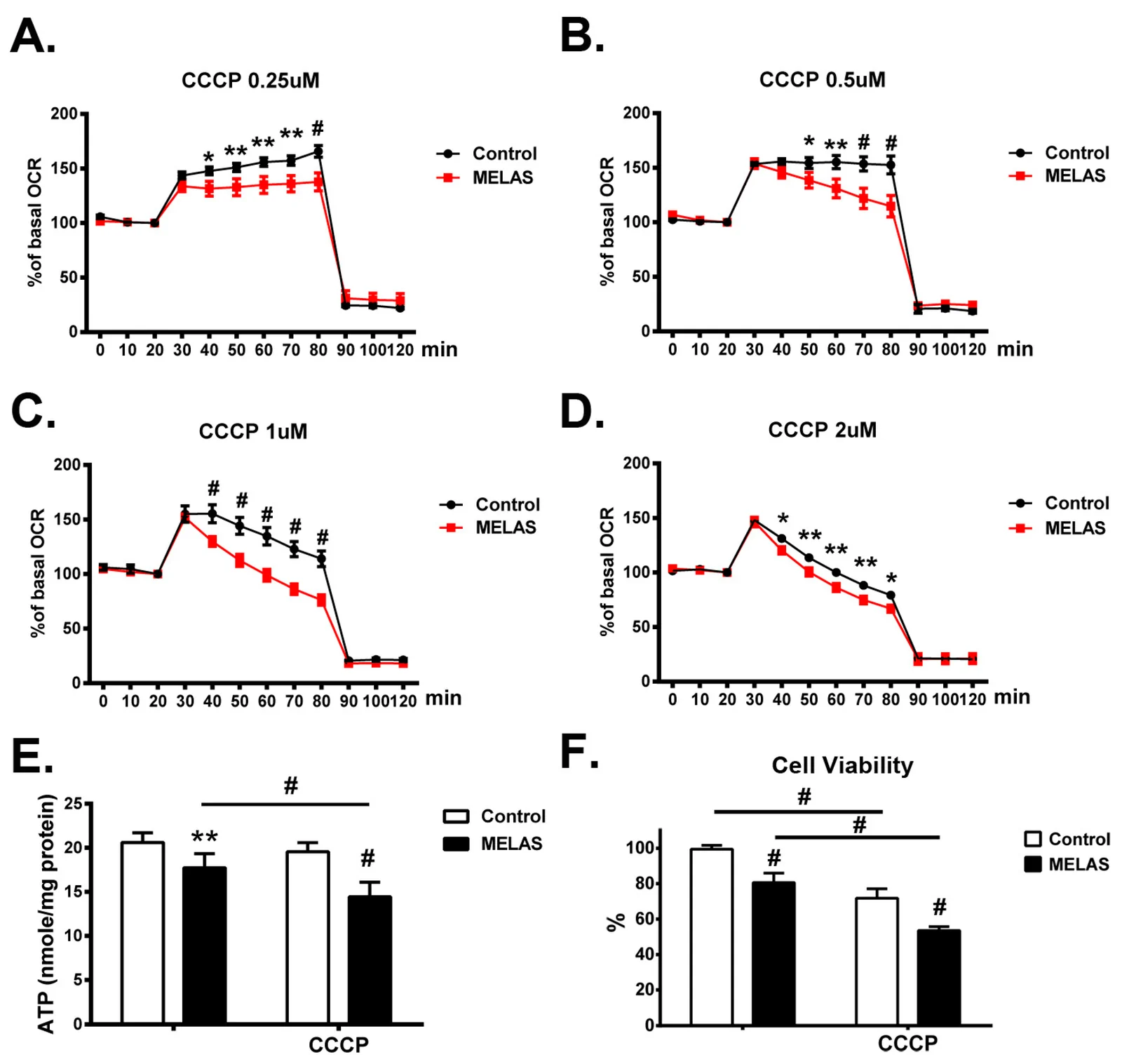
Decreased oxygen consumption rate, ATP production and cell viability. (**A**–**D**)Control cells were able to maintain uncoupled respiration at a higher oxygen consumption rate (OCR) in the presence of CCCP at different concentrations in comparison with MELAS iPS cells. (**E**) Intracellular content of ATP was determined in the presence or absence of CCCP. (**F**) MELAS iPS cells showed a significant decline in cell viability in the presence of CCCP in comparison with control iPS cells. All measurements are expressed as mean values ± SEM, n = 3–5. * *p <* 0.05, ** *p <* 0.01, # *p <* 0.001, versus control.

Real-time assessment of mitochondrial respiration in MELAS iPS cells and its isogenic counterpart (control iPS) was determined by titrating the concentration of protonophore CCCP inducing the maximal oxygen consumption that is subsequently inhibited by rotenone and antimycin A. OCR of MELAS iPS cells remained at a maximal plateau in the presence of 0.25 μM CCCP (Figure 5A), whereas, during the incubation of CCCP from 0.5 to 2 μM (Figure 5B–D), it achieved its maximal level immediately and declined rapidly. On the contrary, the immediate rise and gradual decline from maximal OCR were observed in the control iPS cells upon exposure to a high concentrations (1 μM and 1.5 μM) of CCCP (Figure 5C,D). Overall, the control iPS cells maintained uncoupled respiration at higher OCR, whereas MELAS iPS cells displayed significantly attenuated levels of OCR during the exposure to CCCP at different concentrations. These findings highlighted the impact of OXPHOS deficiency on the mitochondrial function and the declined capacity of mitochondrial respiration upon oxidative insults. 

### 3.10. Deficiency of Intracellular ATP Content

The cellular ATP production of the MELAS iPS cells cultured in basal condition and in the presence of CCCP was determined. Levels of ATP production in the MELAS iPS cells was significantly lower than in the control iPS cells at basal condition (Figure 5E). While in the presence of CCCP, the levels of ATP production in MELAS iPS cells were also significantly lower than in the cells at basal condition, the mean level of ATP production in control iPS cells in the presence of CCCP as compared to basal condition was slightly decreased, though not significantly. These observations suggested the vulnerability of MELAS iPS cells to oxidative stress. 

### 3.11. Decrease of Cell Viability

Excess ROS production has an impact on many cellular biomolecules, including membrane phospholipids, respiratory chain complexes, proteins, and mitochondrial DNA. Consequently, this may lead to cellular dysfunction, and ultimately, cell death. Cell viability upon exposure to CCCP-induced oxidative stress was determined in both control and MELAS iPS cells. Both iPS cells with the same amount were plated in basal culture media for 24 h, then, the cell viability in the presence of CCCP between each iPS cell lines was compared. Both iPS cells lines were vulnerable to CCCP-mediated oxidative stress as shown by the significant decline of cell viability in the presence of CCCP. It should be noted that the cell viability of MELAS iPS cells was consistently significantly lower than that of control iPS cells in the presence of CCCP.

## 4. Discussion

In this work, we demonstrated that aggravation of autophagy dysfunction and mitophagy by CCCP in MELAS iPS cells is contributing to decreased cellular viability in comparison with its normal counterpart. This phenomenon could be explained by the combination of an increased level of ROS, calcium leakage from the intracellular store [26,27], loss of mitochondrial membrane potential, and ultimately the deficiency of energy production. The iPS cells with high heteroplasmy of A3243G mutation showed a deficiency of respiratory complexes I and IV, impairment of respiratory function, attenuated ATP generation, and decreased cell proliferation. These results are all the expected characteristics of fibroblast harboring defects in the OXPHOS system [12]. Furthermore, it has been observed that severe assembly defects and enzyme activities of Complexes I and IV, impaired respiratory function, high level of ROS, low mitochondrial membrane potential, reliance on anaerobic glycolysis, and energy deficiency contribute to the underlying pathogenic mechanism of the MELAS syndrome [30,31]. In this study, we successfully provided iPS cells characterizing the pathogenesis of MELAS syndrome for disease modeling and pathomechanism identification.

With the advantage of retainment of cytoplasmic genetic material during direct reprogramming and variation for mtDNA mutation heteroplasmy during cell passage, isogenic iPS cell clones with high mutant mtDNA burden and without mtDNA mutation could both be isolated simultaneously for observation of the impact of mtDNA heteroplasmy on pathomechanism of mitochondrial diseases [32,33]. In this study, we used isogenic iPS cells with and without high burden of A3243G mutation, and identified an elevated level of autophagy and accumulation of autophagic vesicles in MELAS iPS cells. Recent researches regarding the autophagy in MELAS syndrome have been limited to patient-derived fibroblasts and cybrid cell lines [14,15,16,34]. In the work of Sanchez-Alcazar’s et al., MELAS fibroblasts harboring mtDNA A3243G mutation with 4% to 73% heteroplasmy showed elevated levels of ROS, deficiencies of respiratory complexes I, II, III and IV, dysfunctional mitochondrial activity, impaired autophagic flux, and activation of mitophagy in comparison to normal control cells with different genetic backgrounds [14,15,34]. Of note, a progressive increase in mtDNA A3243G heteroplasmy correlates with the severity of phenotype. Individuals with a lower percentage of A3243G heteroplasmy can face mitochondrial diabetes and autism, and individuals with 90–100% A3243G heteroplasmy are most commonly affected with MELAS syndrome or perinatal lethality [35]. Thus, marked tissue-specific differences and variation of genetic background may modulate the pathogenic expression of the A3243G mutation and the prognosis of mitochondrial diseases [36,37]. To avoid the impact of the genetic background on signal transduction and epigenetic regulation, tumor-derived cytoplasmic hybrid (cybrid) cell lines have been used broadly in the study of mitochondrial diseases [16,35,38]. In a study by Schon’s group, there was no appreciable increase of autophagy in A3243G cybrid cells in the presence or absence of rapamycin compared to that in wild-type cybrid cells, while Sanchez-Alcazar’s group found a significant increase in the ratio of LC3-II to LC3-I in MELAS cybrid cells in comparison with the control cybrid cells [15,16]. Another study further demonstrated a difference in the bioenergetic profile and mitophagy between cybrids derived from adenocarcinoma and rhabdomyosarcoma harboring heteroplasmic A3243G mutation [39]. These findings highlighted the link between genetic background and cell-type dependent regulation of mitochondrial function, and revealed the impact of discrepant results from cell-specific cybrids on the approach for the study of mitochondrial diseases.

In the present study, a higher than normal level of the autophagosomal marker LC3-II was detected in MELAS iPS cells indicating an increase of autophagic flux at the basal condition, while the ATP production was deficient. Previous studies have demonstrated that lower levels of ATP production due to OXPHOS defect in mitochondrial diseases is insufficient for the maintenance of normal cellular function and could trigger autophagy via activation of AMPK and inhibition of mammalian target of rapamycin complex 1 (mTORC1) [12,40,41,42]. Moreover, microscopic observations unveiled a marked increase of enlarged puncta of autophagosomes and autolysosomes in MELAS iPS cells treated with CCCP, suggesting an accumulation of autophagosomes and autolysosomes. Collectively, the enhanced autophagy and accumulation of autophagosomes and autolysosomes implied a disruption of the autophagic flux in MELAS iPS cells upon oxidative stress. These findings are consistent with previous studies reporting that impaired autophagic flux leads to the accumulation of autophagosomes and lysosomes in OXPHOS deficient fibroblasts harboring nuclear DNA mutations [43]. In addition, midbrain of a murine model with mtDNA deletion due to mutant Twinkle demonstrated impairment of mitochondrial respiratory function and increase of autophagy resulting to neurodegeneration, while an accumulation of autophagosomes was observed in the retinal ganglions of murine model of dominant optic atrophy with disrupted mitochondrial dynamics [44,45,46]. It has been indicated that excessive autophagosomes reduces the rate-limiting lysosomal activity and leads to a blockade of autophagy and the accumulation of autophagosomes, further causing a disruption to autophagy and induction of cell toxicity subsequently [47]. 

Our results also demonstrated a marked increase in ROS production, elevation of intracellular calcium, depolarization of mitochondrial membrane potential, and decreased cell viability in MELAS iPS cells following treatment of protonophore CCCP, and suggested that ROS contributes to cell toxicity. It has previously been observed that oxidative stress induces calcium influx from endoplasmic reticulum into the cytoplasm, nuclei, and mitochondria, and leads to disruption of the normal metabolism and signal transduction pathway [48]. Furthermore, mitochondrial calcium overload after a massive calcium flux causes opening of the mitochondrial permeability transition pores resulting in the mitochondrial membrane depolarization, mitochondrial dysfunctions, damages, and ultimately cell death [49,50,51]. Autophagy is induced by mitochondrial depolarization to eliminate the damaged or dysfunctional mitochondria, a specific process called mitophagy that attenuates apoptosis or necrosis [52,53]. Indeed, our results also observed an induction of mitophagy in MELAS iPS cells in the presence of CCCP, whereas mitophagy was scarce at the basal condition, even during a state of enhanced general autophagy. These observations were consistent with previous studies in that mitochondrial defects alone were not sufficient to initiate selective mitophagy in a mitochondrial disease [16]. Moreover, our results further implied that robust collapse of mitochondrial membrane potential and OXPHOS defect synergistically induce mitophagy to eliminate damaged mitochondria. Of note, the levels of Parkin were indistinguishable between control and MELAS iPS cells (unpublished data) in our results, in contrast to previous studies using immortalized cells indicated the initiation of extensive mitophagy upon the combination of macroautophagy and Parkin-mediated mitophagy pathway [16,54]. Our observations were consistent with other studies using neuron cells, which failed to demonstrate Parkin-mediated mitophagy upon enhanced mitochondrial depolarization even with excessive Parkin–mitochondrial localization [55]. Furthermore, another study using fibroblasts showed Parkin recruitment to mitochondria of control cells and OXPHOS defective cells with diminished mitochondrial membrane potential, respectively, without initiation of mitophagy [43]. Given our results and those previously reported, we speculate that additional mechanisms other than Parkin may regulate the initiation of mitophagy. Recently, several studies have identified novel regulators of mitophagy through Parkin-independent pathway for cellular homeostasis [56,57,58]. Thus, further studies analyzing the regulation of mitophagy-related signaling cascades in an iPS cell model of mtDNA mutation are needed to shade more light on the pathological mechanism of mitochondrial diseases.

In conclusion, the iPS cellular model recapitulates the pathogenesis of MELAS syndrome and holds promises for the determination of a pathological mechanism as both the isogenic iPS cells with undetectable-mutation and the high heteroplasmy of mutant mtDNA could be established from parental cells. Of note, mitophagy was scarce in MELAS iPS cells at the basal condition during elevated autophagy; this explained the accumulation and heteroplasmy of pathogenic mtDNA in human patients under the same physiological conditions. Moreover, the combination of the mtDNA mutation and the oxidative insults elicit bulk macroautophagy with an accumulation of autophagosomes and autolysosomes, and leads to the promotion of cell toxicity, activation of mitophagy, and subsequently, the decrease of cell viability. Although there was a limitation of available samples in this patient-specific iPS cells, our results were performed comprehensively and completely in unveiling the role of autophagy in MELAS syndrome. They provided further insights into the autophagy dysfunction and contributed to a better understanding of the pathological mechanism of mitochondrial diseases.
