# Correction: Lin et al. Oxidative Insults and Mitochondrial DNA Mutation Promote Enhanced Autophagy and Mitophagy Compromising Cell Viability in Pluripotent Cell Model of Mitochondrial Disease. *Cells* 2019, *8*, 65

**DOI:** 10.3390/cells15201832

**Published:** 2026-10-09

**Authors:** Dar-Shong Lin, Yu-Wen Huang, Che-Sheng Ho, Pi-Lien Hung, Mei-Hsin Hsu, Tuan-Jen Wang, Tsu-Yen Wu, Tsung-Han Lee, Zo-Darr Huang, Po-Chun Chang, Ming-Fu Chiang

**Affiliations:** 1 Department of Pediatrics, Mackay Memorial Hospital, Taipei 10449, Taiwan; 2 Department of Medicine and Institute of Biomedical Sciences, Mackay Medical College, New Taipei 25245, Taiwan; 3 Department of Medical Research, Mackay Memorial Hospital, Taipei 10449, Taiwan; 4 Department of Pediatric Neurology, Kaohsiung Chang Gung Memorial Hospital, and Chang Gung University College of Medicine, Kaohsiung 88301, Taiwan; 5 Department of Laboratory Medicine, Mackay Memorial Hospital, Taipei 10449, Taiwan; 6 Department of Information Technology, Mackay Memorial Hospital, Taipei 10449, Taiwan; 7 Department of Neurosurgery, Mackay Memorial Hospital, Taipei 10449, Taiwan; 8 Mackay Medicine, Nursing and Management College, Taipei 11260, Taiwan; 9 Graduate Institute of Injury Prevention and Control, Taipei Medical University, Taipei 11031, Taiwan

## Error in Figure 1A

In the original publication [1], there was a mistake in Figure 1A (Oct4, Control and MELAS) as published. There was overlap between the Oct4 immunofluorescence images presented for the Control and MELAS iPSC groups due to an error during figure assembly. The affected panel has been replaced with the corresponding correct image, and the corrected Figure 1A appears below. The authors state that the scientific conclusions are unaffected. This correction was approved by the Academic Editor. The original publication has also been updated.

## Error in Figure 3A

In the original publication, there was a mistake in Figure 3A (MitoSOX Red, Basal, Control and MELAS) as published. There was overlap between the MitoSOX Red fluorescence images presented for the Control and MELAS iPSC groups under basal conditions due to an error during figure assembly. The affected panels have been replaced with the corresponding correct images, and the corrected Figure 3A appears below.

## Figures and Tables

**Figure 1:**
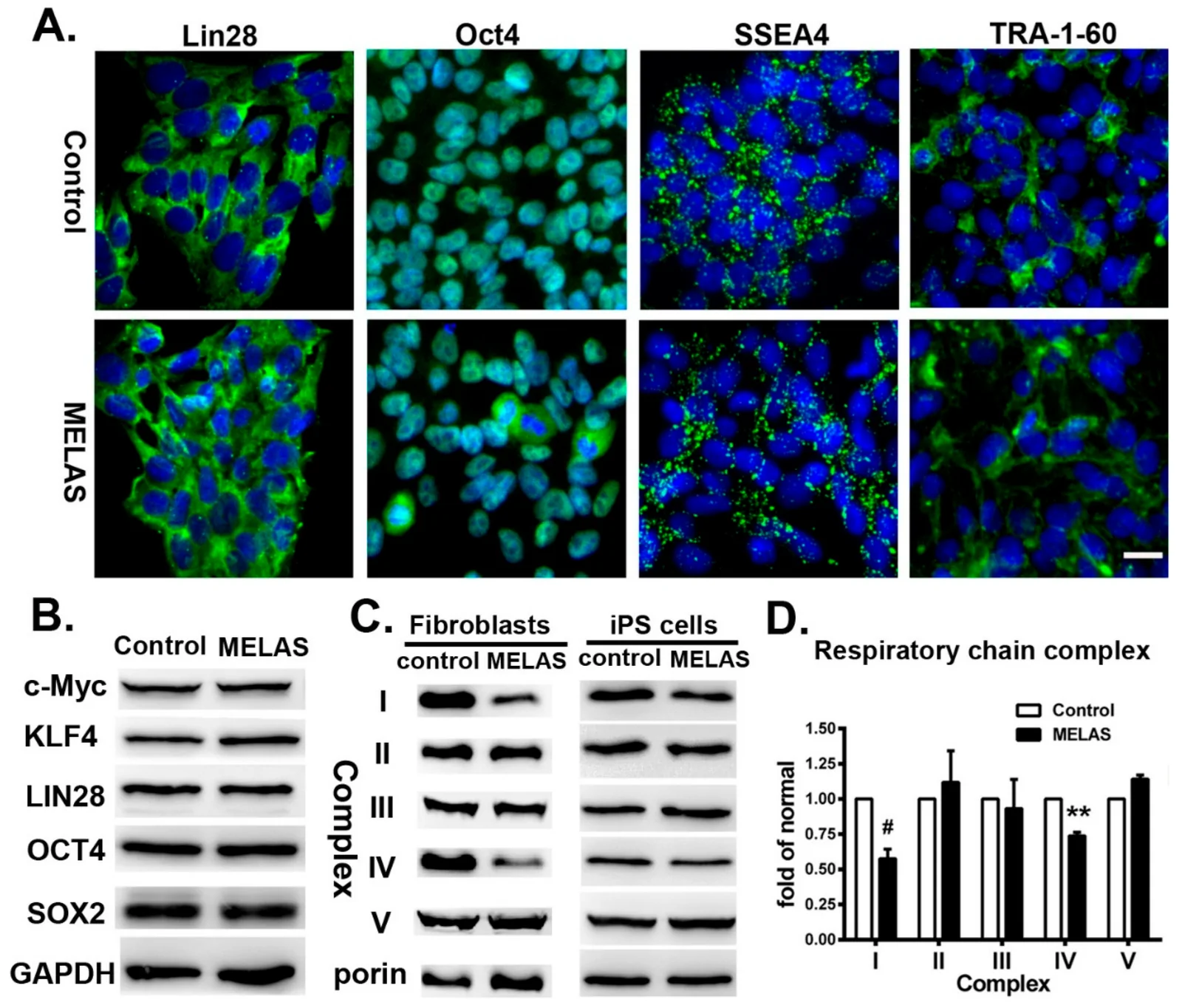
Induced pluripotent stem (iPS) cells derived from fibroblasts of mitochondrial encephalomyopathy, lactic acidosis, and stroke-like episodes (MELAS) syndrome patient. (**A**) Immunocytochemical analyses showed positive expression of pluripotency markers Lin28, Oct4, SSEA4 and Tra-1-60 on both control and MELAS iPS cells. (**B**) Western blots showing the expression of c-Myc, Klf4, Lin28A, Oct4, Sox2 in iPS cells. (**C**) Western blots showing the expression of respiratory complexes in fibroblasts and iPS cells, respectively. (**D**) Quantification of respiratory chain complex proteins in iPS cells represented as fold of normal. All measurements are expressed as mean values ± SEM, n = 3–5. Comparisons with the control cells were performed by the ANOVA-test. ** *p* < 0.01, # *p* < 0.001, versus control. Scale bar: 20 μm.

**Figure 3:**
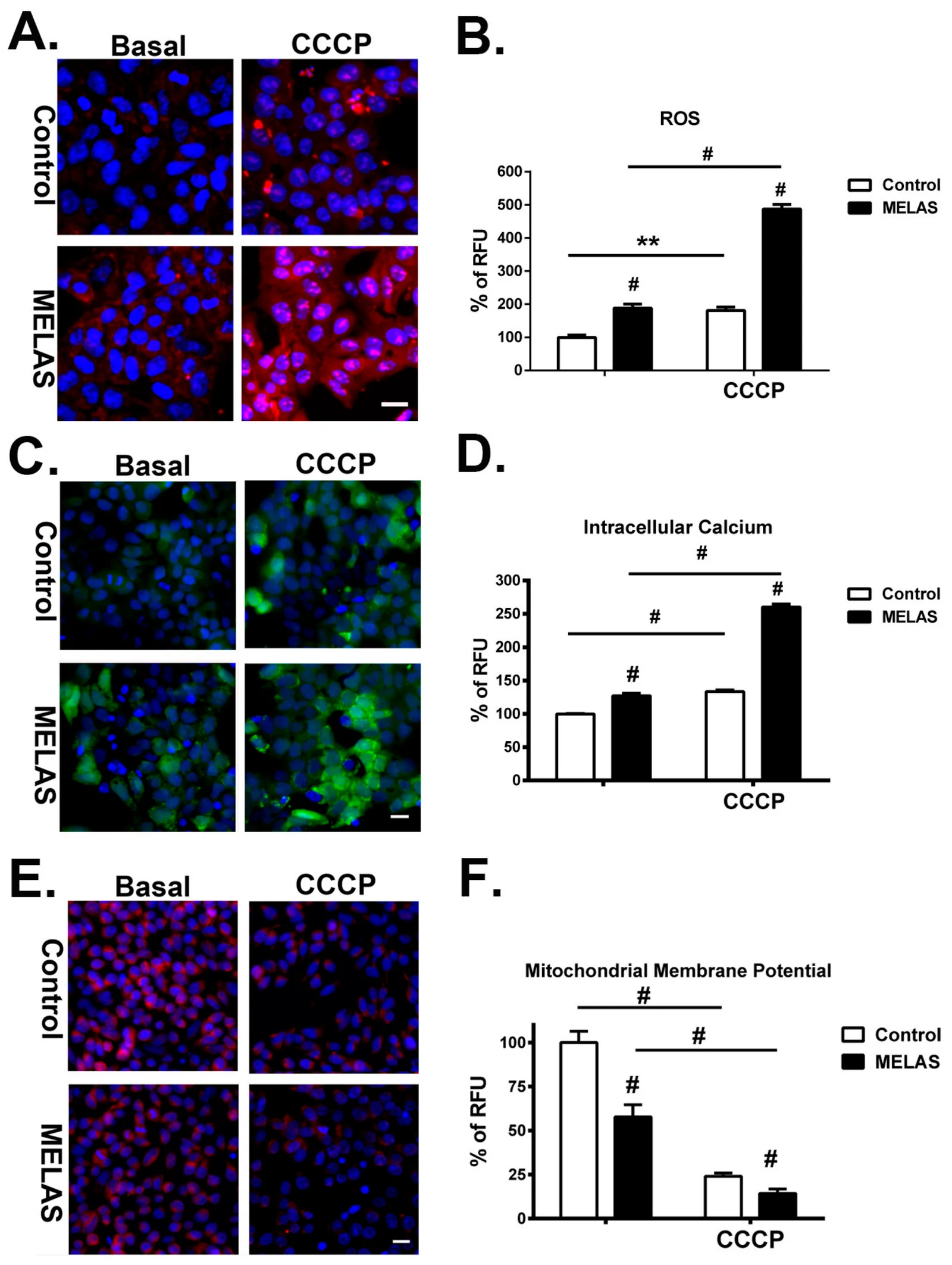
Increase of reactive oxygen species (ROS), intracellular calcium, and mitochondrial depolarization in MELAS iPS cells. (**A**) Representative microscopy fluorescence images showing levels of ROS by staining with MitoSox Red in iPS cells in the presence or absence of CCCP. (**B**) Quantification of ROS levels by analyzing the fluorescence intensity of MitoSox Red. (**C**) Representative microscopy fluorescence images showing levels of intracellular calcium by staining with calcium-sensitive fluorescent dye, fluo-8, in iPS cells in the presence or absence of CCCP. (**D**) Quantification of intracellular calcium levels by analyzing the fluorescence intensity of fluo-8. (**E**) Microscopy fluorescence images showing levels of mitochondrial depolarization by staining with tetramethylrhodamine ethyl ester (TMRE) in iPS cells in the presence or absence of CCCP. (**F**) Quantification of mitochondrial depolarization levels by analyzing the fluorescence intensity of TMRE. All measurements, normalized for number of cells, are expressed as mean values ± SEM, n = 3–5. ** *p* < 0.01, # *p* < 0.001, versus control. RFU: relative fluorescence unit. Scale bar: 20 μm.
